# Um Achado Acidental de um Sarcoma Cardíaco

**DOI:** 10.36660/abc.20210703

**Published:** 2022-07-29

**Authors:** Rita Reis Santos, João Abecasis, Daniel A. Gomes, Mariana Sousa Paiva, Bruno Rocha, Regina Ribeiras, Pedro Freitas, Miguel Abecasis, Marisa Trabulo

**Affiliations:** 1 Centro Hospitalar de Lisboa Ocidental Hospital de Santa Cruz Carnaxide Portugal Hospital de Santa Cruz, Centro Hospitalar de Lisboa Ocidental, Carnaxide – Portugal; 2 Hospital Lusíadas Lisboa Portugal Hospital Lusíadas, Lisboa – Portugal; 3 Nova Medical School Lisboa Portugal Nova Medical School, Lisboa – Portugal

**Keywords:** Neoplasias Cardíaca, Sarcoma, Fibrilação Atrial

## Introdução

Os tumores cardíacos primários são raros e sua apresentação clínica varia desde a descoberta incidental em exames de imagem até apresentações com risco de vida.^1-3^

Relatamos o caso de uma jovem do sexo feminino com história de fibrilação atrial (FA) em quem foi encontrada uma massa em átrio esquerdo em tomografia computadorizada realizada antes da ablação da FA e qualificada para ressecção cirúrgica da massa. O exame anatomopatológico revelou sarcoma cardíaco pleomórfico primário indiferenciado.

## Relato de caso

Paciente do sexo feminino, 49 anos, caucasiana, com história prévia de FA paroxística, encaminhada para ablação eletiva de FA em quem foi identificada incidentalmente uma massa em átrio esquerdo (AE) em tomografia computadorizada cardíaca pré-intervenção (TCC). Ela não tinha sintomas relacionados à massa.

Na avaliação inicial, o eletrocardiograma (ECG) estava em ritmo sinusal. O exame físico e a análise laboratorial completa não revelaram achados anormais.

Além da história de FA paroxística, diagnosticada dois anos antes, a paciente era saudável. As medicações à admissão eram edoxabano (nas últimas 2 semanas antes da ablação), flecainida e bisoprolol, com controle subótimo do ritmo.

### Investigações

**Figura 1:**
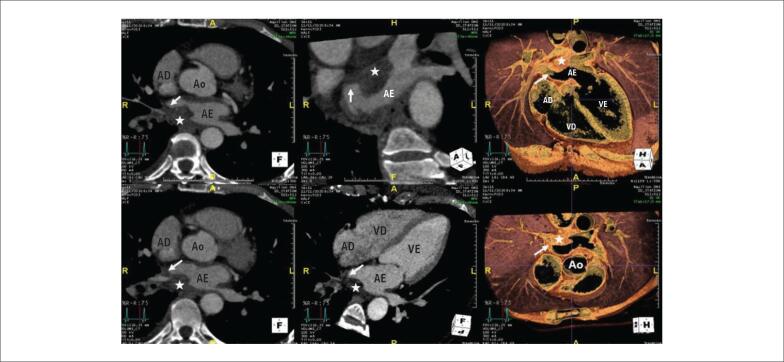
Tomografia computadorizada cardíaca. Uma massa séssil (*) homogênea, hipodensa e levemente irregular, localizada na parede posterior do átrio esquerdo (AE), envolvendo ambos os óstios das veias pulmonares direitas (setas). Ao: aorta; AD: átrio direito; VD: ventrículo direito; VE: ventrículo esquerdo.

Uma massa séssil homogênea, hipodensa, levemente irregular, localizada na parede posterior do AE, envolvendo ambos os óstios das veias pulmonares direitas (VPD), estava presente na TCC (Figura 1). Após esse achado, a paciente realizou um estudo imagológico completo.

**Figura 2:**
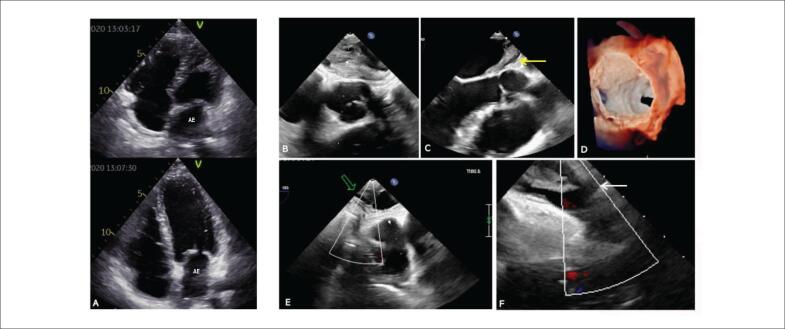
A) Estudo ecocardiográfico transtorácico mostrando massa densa e irregular espessada no teto do AE próximo aos óstios das veias pulmonares direitas; B – D) Ecocardiograma transesofágico mostrando massa isoecogênica estendendo-se da parede posterior da raiz da aorta em direção à parede posterior do átrio esquerdo; anteriormente há um plano de clivagem entre a massa e a parede atrial esquerda (seta amarela); posteriormente este não é o caso, pois os limites da massa não são claramente identificados. E - F) Ecocardiograma transesofágico mostrando massa do AE relacionada às veias pulmonares direitas (veia pulmonar inferior direita (VIP) e veia pulmonar superior direita (VPUP)). E) Compressão do fluxo de saída de RIPV conforme mostrado por moldagem ostial (seta verde). F) Sem interferência na vazão de RUPV (seta branca). AE: átrio esquerdo.

O estudo ecocardiográfico transtorácico revelou uma massa densa, irregular e espessada no teto do AE próximo à entrada das VPD (Figura 2A). O ecocardiograma transesofágico mostrou massa volumosa no AE estendendo-se para a parede posterior da raiz da aorta e apêndice do AE com aparente plano de clivagem anterior entre a massa e a parede do AE (Figura 2B a 2D).

**Figura 3:**
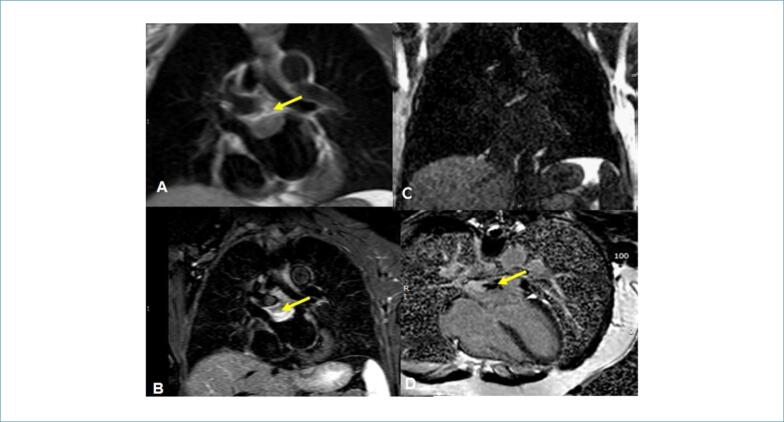
Estudo de RMC. Massa AE (setas amarelas). A) Sequência ponderada em T1 mostrando uma massa isointensa na parede do átrio esquerdo. B) sequência ponderada em T2 correspondente com supressão de gordura mostrando alta intensidade de sinal em toda a massa. C) perfusão de primeira passagem com captação de contraste. D) sequência de realce tardio com aspecto heterogêneo positivo para presença de fibrose.

Na ressonância magnética cardíaca (RMC), uma massa claramente definida com um diâmetro máximo de 32 mm foi mostrada. Esta era isointensa nas sequências ponderadas em T1, brilhante nas sequências ponderadas em T2, com alguma perfusão heterogênea e realce tardio com gadolínio (Figura 3).

### Diagnóstico diferencial

Suspeitou-se inicialmente de mixoma por ser a massa incidental assintomática mais comum, além do trombo, com origem no átrio esquerdo. Neste caso, a ausência de pedículo, as características imagológicas e a sua localização específica ao longo da parede posterior e superior do AE levantaram a suspeita de outra entidade clínica, nomeadamente com possível comportamento maligno.

### Gerenciamento

Dada a suspeita de malignidade, um F-18 FDG PET/CT foi realizado para identificar tanto o tumor primário quanto as possíveis lesões associadas à distância. Foi identificada uma massa solitária em átrio esquerdo. Realizou-se TC de corpo inteiro para estadiamento, que foi negativa para doença extracardíaca.

Diante do diagnóstico presuntivo de tumor cardíaco primário de comportamento potencialmente maligno, seja por extensão local ou risco embólico, optou-se por proceder à ressecção cirúrgica para fins diagnósticos e terapêuticos. No intraoperatório, a massa ressecada estendeu-se pela parede posterior do AE até a comissura anterior do anel mitral, sem infiltração das veias pulmonares. A infiltração posterior da massa com descolamento incompleto forçou a necessidade de reconstrução do septo atrial e interatrial usando um segmento de pericárdio. A recuperação pós-operatória transcorreu sem intercorrências.

**Figura 4:**
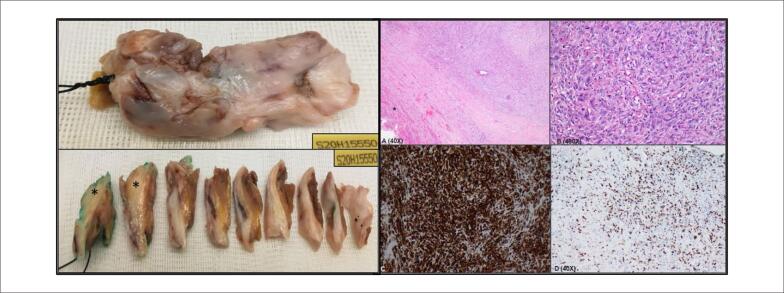
Achados macroscópicos: massa elástica irregular de 50x25x10mm. A superfície de corte apresentava uma massa infiltrativa e fibrosa (*) sem necrose evidente ou alterações mixóides. A (H&E): Borda do tumor com miocárdio saudável (*); B (H&E): Grande aumento mostrando áreas sólidas com fina rede de colágeno reticulado e pleomorfismo nuclear moderado, nucléolos focais proeminentes e mitose; C – D: Estudo imuno-histoquímico – positivo para vimentina e Ki67 (com ~40% de positividade), respectivamente.

A avaliação macroscópica revelou um fragmento elástico irregular de 50x25x10 mm (Figura 4). O exame histopatológico à coloração de hematoxilina e eosina mostrou que a lesão era composta por áreas sólidas com fina rede reticulada de colágeno e pleomorfismo nuclear moderado com nucléolos focais proeminentes e atividade mitótica (característica de um sarcoma pleomórfico indiferenciado) (Figura 4B). A imunohistoquímica revelou positividade para vimentina, um marcador mesenquimal (Figura 4C) com marcadores epiteliais e musculares negativos. Ki67 foi 40% positivo em áreas mais proliferativas (Figura 4D). Assumiu-se o diagnóstico de sarcoma cardíaco pleomórfico primário indiferenciado com possível amplificação de MDM2.

A paciente recebeu alta no nono dia após a cirurgia. Ela foi encaminhada para terapia adjuvante. No quinto mês de seguimento já tinha completado 2 ciclos de quimioterapia com doxorrubicina e ifosfamida e encontra-se agora fazendo radioterapia com evolução clínica sem intercorrências.

## Discussão

Os tumores primários do coração são entidades extremamente raras, com incidência inferior a 0,1 por cento.^1,2^ São tumores agressivos que podem ser sintomáticos ou, se não produzirem sintomas até que estejam localmente avançados, como no caso relatado, são encontrados incidentalmente durante um estudo de imagem cardíaca.^2,3^ Costumam ocorrer em pacientes jovens com idade média de 44 anos e estão distribuídos aproximadamente igualmente entre os sexos.^4^ Os sarcomas cardíacos, embora extremamente raros, são as lesões malignas primárias mais comuns.^5,6^ Dependendo do subtipo, podem surgir de células mesenquimais de ventrículos, átrios ou pericárdio. Essas malignidades proliferam rapidamente e causam a morte por infiltração generalizada do miocárdio, obstrução dos principais vasos cardíacos e/ou metástases à distância.^7^

A ressecção cirúrgica é o tratamento local mais eficaz para sarcomas cardíacos, principalmente em pacientes com doença não metastática.^8,9^ Embora os sarcomas cardíacos são tumores altamente invasivos, margens cirúrgicas claras são difíceis de obter e, portanto, podem recorrer facilmente, destacando a necessidade de tratamentos locais e sistêmicos mais eficazes que podem ser usados em conjunto com a cirurgia para melhorar os resultados dos pacientes.^8-10^ A sobrevida média ao diagnóstico é de 6 a 12 meses, mesmo após a excisão cirúrgica completa.^2,4^

## Conclusões

As neoplasias cardíacas primárias são entidades muito raras, sendo anedóticas como achados assintomáticos. O local de inserção e algumas características de imagem, apenas totalmente detalhadas sob avaliação multimodal, fornecem pistas para o diagnóstico diferencial, nomeadamente com lesões benignas mais comuns. O uso de ferramentas avançadas de imagem para estadiamento é fundamental na definição da estratégia de tratamento mais adequada. A ressecção cirúrgica completa é solicitada como a primeira opção.
