# Supplementary material for: Um Achado Acidental de um Sarcoma Cardíaco
Source: Arq Bras Cardiol. 2022 Jul 29;119(2):349–52. [Article in Portuguese] doi: 10.36660/abc.20210703 (PMC9363064; doi:10.36660/abc.20210703)
Supplement: Translation

# Translation: An Incidental Finding of a Cardiac Sarcoma

**Authors:** Rita Reis Santos, João Abecasis, Daniel A. Gomes, Mariana Sousa Paiva, Bruno Rocha, Regina Ribeiras, Pedro Freitas, Miguel Abecasis, Marisa Trabulo

**Affiliations:** 1 Carnaxide Portugal Hospital de Santa Cruz, Centro Hospitalar de Lisboa Ocidental, Carnaxide – Portugal; 2 Lisboa Portugal Hospital Lusíadas, Lisboa – Portugal; 3 Lisboa Portugal Nova Medical School, Lisboa – Portugal

**Keywords:** Heart Neoplasms, Sarcoma, Atrial Fibrillation

## Introduction

Primary cardiac tumors are rare, and their clinical presentation ranges from incidental discovery on imaging tests to life-threatening presentations.^1-3^

We report a case of a young female with a history of atrial fibrillation (AF) who was found a left atrial mass in an elective cardiac computed tomography performed before AF-ablation and was qualified for surgical resection of the mass. A pathological exam revealed a primary undifferentiated pleomorphic cardiac sarcoma.

## Case report

A 49 year-old Caucasian female with a previous history of paroxysmal AF, referred for elective percutaneous AF-catheter ablation, was referred to the hospital due to a left atrial (LA) mass incidentally identified at pre-interventional cardiac computed tomography (CCT). She had no symptoms related to the mass.

On initial evaluation, an electrocardiogram (ECG) was in sinus rhythm. Physical examination and full laboratory analysis revealed no abnormal findings.

Beyond the history of paroxysmal AF, diagnosed two years before, the patient was otherwise healthy. The medications on admission were edoxaban (in the last 2 weeks before ablation), flecainide and bisoprolol, with sub-optimal rhythm control.

### Investigations

A homogenous, hypodense, slightly irregular sessile mass localized in the posterior wall of the LA, involving both ostia of the right pulmonary veins (RPV), was present in CCT (Figure 1). After this finding, the patient performed a complete imagological study.

**Figure 1:** Cardiac computed tomography. A homogenous, hypodense, slightly irregular sessile mass (*), located in the posterior wall of the left atrium (LA), involving both ostia of the right pulmonary veins is depicted (arrows). Ao: Aorta; RA: Right atrium; RV: Right Ventricle; LV: Left Ventricle.

The transthoracic echocardiographic study revealed a dense, irregular, thickened ceiling mass of the LA roof near to RPV entrance (Figure 2A). Transesophageal echocardiography showed voluminous LA mass extending into the posterior wall of the aortic root and LA appendage with an apparent anterior cleavage plane between the mass and LA wall (figure 2B to 2D).

**Figure 2:** A) Transthoracic echocardiographic study showing a dense and irregular thickened ceiling mass of the LA near the right pulmonary veins ostia; B – D) Transesophageal echocardiogram showing an isoecogenic mass extending from the posterior wall of the aortic root towards the posterior left atrial wall; anteriorly there is a cleavage plane between the mass and left atrial wall (yellow arrow); posteriorly this is not the case as the limits of the mass are not clearly identified. E - F) Transesophageal echocardiogram showing LA mass related to the right pulmonary veins (right inferior pulmonary vein (RIPV) and right upper pulmonary vein (RUPV)). E) RIPV outflow compression as showed by ostial molding (green arrow). F) No interference with the RUPV outflow (white arrow). LA: left atrium.

At cardiac magnetic resonance (CMR) study, a clearly defined mass with a maximum 32 mm diameter was shown. This was isointense at T1-weighed sequences, bright at T2-weighed sequences, with some heterogeneous perfusion and late gadolinium enhancement (Figure 3).

**Figure 3:** CMR study. LA mass (yellow arrows). A) T1-weighed sequence depicting an isointense posterior left atrium wall mass. B) corresponding T2-weighed sequence with fat-suppression showing high signal intensity across the mass. C) first-pass perfusion with contrast uptake. D) delayed enhancement sequence with a heterogeneous aspect positive for the presence of fibrosis.

### Differential diagnosis

A myxoma was initially suspected as it represents the most common asymptomatic incidental mass, beyond thrombus, arising in the left atrium. In this case, the absence of a pedicle, several imagological features and its specific location along the posterior and superior LA wall raised the suspicion of another clinical entity, namely possible malignant behavior.

### Management

Given the suspicion of malignancy, an F-18 FDG PET/CT was performed to identify both primary tumor and potential distant associated lesions. A solitary left atrial mass was identified. A complete body CT scan was performed for staging purposes, and it was negative for extra-cardiac disease.

Given the presumptive diagnosis of a primary cardiac tumor of potential malignant behavior, either for local extension or embolic risk, a decision was made to proceed with surgical resection for diagnostic and treatment purposes. Intraoperatively, the resected mass extended through the posterior wall of LA until the anterior commissure of the mitral annulus, without pulmonary veins infiltration. Posterior infiltration with incomplete mass detachment forced the need for both atrial and inter-atrial septum reconstruction using a pericardial patch. Postoperative recovery was uneventful.

The macroscopic evaluation revealed an irregular elastic fragment of 50x25x10 mm (Figure 4). Histopathological examination on hematoxylin and eosin stain showed that the lesion was composed of solid areas with a fine reticulate network of collagen and moderate nuclear pleomorphism with prominent focal nucleoli and mitotic activity (characteristic of an undifferentiated pleomorphic sarcoma) (Figure 4B). Immunohistochemistry revealed positivity for vimentin, a mesenchymal marker (Figure 4C) with negative epithelial and muscular markers. Ki67 was 40% positive in more proliferative areas (Figure 4D). The diagnosis of a primary undifferentiated pleomorphic cardiac sarcoma with possible MDM2 amplification was assumed.

**Figure 4:** Macroscopic findings: 50x25x10mm irregular elastic mass. The cut surface showed an infiltrative and fibrous mass (*) without evident necrosis or myxoid changes. A (H&E): Tumor border with healthy myocardium (*); B (H&E): High power showing solid areas with a fine reticulate collagen network and moderate nuclear pleomorphism, prominent focal nucleoli, and mitosis; C – D: Immunohistochemistry study – positive for both vimentin and Ki67 (with ~40% positivity), respectively.

The patient was discharged on the ninth day after surgery. She was referred for adjuvant therapy. At the fifth month of follow-up, she had already completed 2 cycles of chemotherapy with doxorubicin and ifosfamide and is now performing radiotherapy with an uneventful clinical course.

## Discussion

Primary tumors of the heart are extremely rare entities, with less than 0.1 percent incidence.^1,2^ They are aggressive tumors that may be symptomatic or, if they do not produce symptoms until they are locally advanced, as in the case reported, they are found incidentally during a cardiac imaging study.^2,3^ They tend to occur in young patients with a mean age of 44 years and are approximately equally distributed between the sexes.^4^ Cardiac sarcomas, albeit extremely rare, are the most common primary malignant lesions.^5,6^ Depending on the subtype, they can arise from mesenchymal cells of ventricles, atria or pericardium. These malignancies proliferate quickly and cause death through widespread infiltration of the myocardium, obstruction of major cardiac vessels, and/or distant metastases.^7^

Surgical resection is the most effective local treatment for cardiac sarcomas, mainly in patients with nonmetastatic disease.^8,9^ Although cardiac sarcomas are highly invasive tumors, clear surgical margins are difficult to obtain, and so they can easily recur, highlighting the need for more effective local and systemic treatments that may be used in conjunction with surgery to improve patient outcomes.^8-10^ Median survival at diagnosis is 6 to 12 months even after complete surgical excision.^2,4^

## Conclusions

Primary cardiac malignancies are very rare entities, being anecdotal as asymptomatic findings. Insertion location and some imaging features, only fully detailed under multimodality assessment, provide clues toward differential diagnosis, namely with more common benign lesions. The use of advanced imaging tools for staging is key when defining the most appropriate treatment strategy. Complete surgical resection is prompted as the first option.
